# The SRC/NF‐κB‐AKT/NOS3 axis as a key mediator of Kaempferol's protective effects against oxidative stress‐induced osteoclastogenesis

**DOI:** 10.1002/iid3.70045

**Published:** 2024-10-18

**Authors:** Jiaming Shen, Chunjie Hu, Yuelong Wang, Yiying Tan, Xiaochen Gao, Nanxi Zhang, Jingwei Lv, Jiaming Sun

**Affiliations:** ^1^ Jilin Ginseng Academy Changchun University of Chinese Medicine Changchun China; ^2^ Affiliated Hospital Changchun University of Chinese Medicine Changchun China

**Keywords:** Kaempferol, osteoclasts, osteoporosis, oxidative stress, SRC/NF‐κB‐AKT/NOS3 axis

## Abstract

**Background:**

Osteoclasts are integral to the advancement of osteoporosis (OP), and their generation under conditions of oxidative stress (OS) involves various pathways. However, the specific mechanism through which the natural antioxidant kaempferol (KAE) mitigates the influence of OS on osteoclasts remains somewhat uncertain. This study aims to evaluate the effect of KAE on osteoclast formation under OS and explore its possible mechanism.

**Methods:**

Zebrafish were used to observe the effects of KAE on OP and OS. OP and OS "double disease targets" network pharmacology were used to predict the action target and mechanism of KAE on OP under OS. The effects of KAE on osteoclast differentiation induced by OS were evaluated using RWA264.7 cells induced by LPS. To elucidate the potential mechanism, we detected the expression of related factors and target genes during induction.

**Results:**

The presence of KAE exhibited potential in improving the conditions of OP and OS in zebrafish. KAE can reduce the OS of RAW 264.7 cells stimulated by LPS, inhibit the formation of osteoclasts, and change the level of related factors of OS, and reduce the increase of TRAP. The utilization of network pharmacology and target gene expression assay revealed that KAE exerted a down‐regulatory effect on the expression of proto‐oncogene tyrosine protein kinase (SRC), nuclear factor kappa‐B (NF‐κB), Serine/Threonine Kinase‐1 (AKT1), Nitric Oxide Synthase 3 (NOS3) and Matrix Metallopeptidase‐2 (MMP2).

**Conclusion:**

Based on the results of this study, KAE may effectively mitigate OS and impede the formation of osteoclasts through the SRC/NF‐κB‐AKT/NOS3 axis.

## Graphical abstract

This work demonstrates that kaempferol inhibits osteoclast formation by inhibiting the SRC/NF‐κB‐AKT/NOS3 axis. It is suggested that kaempferol can treat op by reducing oxidative stress and inhibiting osteoclasts. The results of this study explain the possible mechanism of kaempferol on osteoclasts and provide a new way for kaempferol to treat osteoporosis.

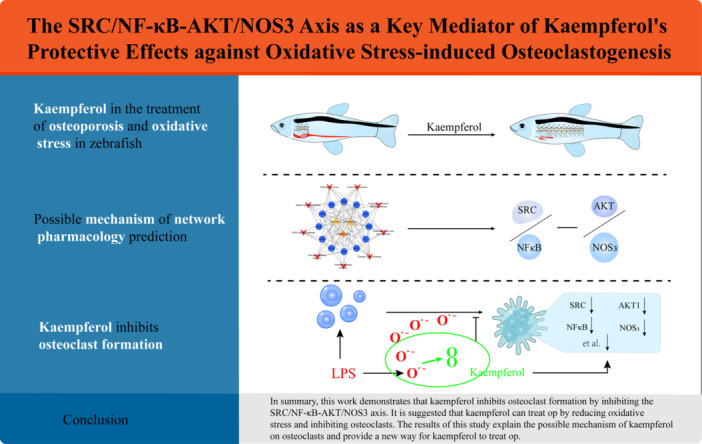

## 1 INTRODUCTION

OP has garnered significant attention in the realm of human health due to its pervasive impact and escalating prevalence.^1^, ^2^ OP arises from an imbalance in bone homeostasis, primarily manifesting as a reduction in bone mass, heightened bone fragility resulting from compromised bone tissue microstructure, and an increased susceptibility to fractures.^3^, ^4^ The maintenance of bone homeostasis is achieved through the equilibrium between osteogenesis and bone resorption, which is facilitated by the actions of osteoblasts and osteoclasts. Osteoblasts originate from mesenchymal bone progenitor cells and play a key role in bone formation. It is responsible for secreting a variety of extracellular matrix proteins, including type I collagen, osteopontin, osteocalcin and alkaline phosphatase.^5^ In addition, they also secrete receptor activators of macrophage colony stimulating factor (M‐CSF) and nuclear factor B (RANK) ligand (RANKL) to stimulate monocytes in hematopoietic stem cell lineage to differentiate into osteoclasts and cause bone resorption.^6^ Therefore, balancing the function of osteoblasts and osteoclasts is the key to the treatment of OP.^4^

The induction of OS has been demonstrated to disrupt bone homeostasis.^7^ OS is the result of an overabundance of highly reactive molecules, like reactive oxygen species (ROS).^8^ The excessive production of ROS has been strongly associated with the initiation and advancement of OP. Studies have provided evidence that OS can negatively affect osteoblasts by inhibiting the Wnt/β‐catenin signaling pathway and diminishing the expression of β‐catenin.^9^ Excessive ROS acts as an endogenous medium for osteoclast differentiation to promote osteoclast formation.^10^ Simultaneously, the rise in ROS resulted in alterations in the quantities of proinflammatory agents and miRNA, thereby augmenting the creation of osteoclasts and reducing the production of osteoblasts via the activation mechanisms of MAPK and transcription factors.^9^ Therefore, inhibition of OS and osteoclast production and activation is an effective means for the treatment of OP.

KAE is a natural flavonoid antioxidant found in many plants, including Chinese herbal medicine and crops.^11^, ^12^ It is an important flavonoid in the diet, accounting for 22‐29% of total flavonoid intake.^13^ Previous studies have revealed the diverse pharmacological attributes of KAE, encompassing its anticancer, anti‐inflammatory, antioxidative, cardioprotective, and anti‐osteoporotic properties.^14^, ^15^, ^16^, ^17^, ^18^ Xie and colleagues discovered that KAE can improve the inhibitory effect of dexamethasone on osteogenesis of MC3T1‐E38 cells or improve OP by increasing the expression of CCCL12 to promote osteoblast differentiation of BMSC.^17^, ^19^ By reducing OS, KAE can also enhance mineralization and stimulate the secretion of OPG in MC3T3‐E1 cells.^20^ However, the effect and mechanism of KAE on osteoclast formation by inhibiting OS have not been fully clarified.

Network pharmacology offers the benefits of multivariate combination and comprehensive analysis by systematically examining the intricate interplay among diseases, compounds, and targets.^21^ Consequently, it can be used to predict the potential mechanism of the effect of components on disease. On the other hand, molecular dynamics simulations furnish valuable insights into unraveling the functional mechanisms of proteins and other biomolecules.^22^ And zebrafish have a high degree of homology with humans.^23^ Compared with ovariectomy in mice or rats, the establishment of zebrafish OP models is simpler and more convenient Its embryos are transparent and easy to observe, and the experimental period is short.^24^ It can simulate various causes of OP such as OS,^25^, ^26^ and is widely used in experimental research on OP. Hence, this study aims to utilize network pharmacology to predict the potential targets of KAE in combating OP and OS, while also employing KEGG enrichment to analyze the plausible action mechanism. The therapeutic effects of KAE on OP and OS were investigated using zebrafish. Additionally, the impact of KAE on osteoclast formation and its underlying mechanism under OS were examined. The findings from this research endeavor will contribute to a better understanding of the specific mechanism of KAE against OP.

## 2 MATERIALS AND METHODS

### 2.1 Materials and chemicals

KAE was acquired from Shanghai Yuanye Biotechnology Co., Ltd. (LOT: 110861‐202303), (the purity ≥99%). The DMEM culture medium (LOT: C11995500BT), fetal bovine serum (LOT: 10099‐141), penicillin (100 U/mL) and streptomycin (100 µg/mL) (LOT: 15140122) were obtained from GIBCO. Ascorbic acid (LOT: 20006621), dimethyl sulfoxide (DMSO) (LOT: WXBD4591V), Calcein (LOT: 027909), dexamethasone (LOT: D8041, the purity ≥99%), β‐glycerophosphate (LOT: G5422‐1), 2,2′‐Azodiisobutyramidine dihydrochloride (AAPH) (LOT: C13116328), prednisolone (LOT: T20J7H9291), 2,7‐difluorofluorescein diacetate (DCFH‐DA) (LOT: P2033707) and etidronate disodium (ED) (LOT: 101174‐201001) were obtained from Beijing Chemical Factory Co., Ltd. The Tartrate‐resistant acid phosphatase (TRAP) was acquired from Beyotime Biotechnology Co., Ltd. (LOT: 051023230918). Lipopolysaccharide (LPS) was obtained from Beijing Solebao Technology Co., Ltd. (LOT: 324R031). A 4% paraformaldehyde solution was purchased from Yuanye Biotechnology Co., Ltd., with the batch number J08IR17105A. The TNF‐α, SOD, NO, GSH, MAD, and IL‐6 enzyme‐linked immunosorbent assay kit were procured from Shanghai enzyme‐linked Biotechnology Co., Ltd. (LOT: mL862859‐J, J2389‐A, mL039693‐J, J2658‐A, J9264‐A, 20230803‐30219 A).

### 2.2 Experimental cells and animals

The wild‐type zebrafish were sourced from Nanjing EzeRinka Biotechnology Co., Ltd. in China. The animal study protocol was approved by the Laboratory Animal Ethics Committee of Changchun University of Chinese Medicine (IACUC approval numbers: 2021519, date of approval: 19 November 2021) for studies involving animals. The RAW 264.7 cell line, derived from mouse monocyte/macrophage cells, was acquired from the Shanghai Cell Bank of the Chinese Academy of Sciences in Shanghai, China.

### 2.3 Breeding and cultivation of zebrafish

Zebrafish of the wild‐type AB variety were raised individually based on their gender in fish tanks that were equipped with lighting and heating mechanisms. The temperature of the water in the cylinder is maintained at approximately 26.5°C, while the pH is regulated within the range of 7–8. Additionally, the light cycle is a 14‐h light/10‐h dark cycle. Feed the harvest shrimp larvae (shrimp eggs are filtered after 24 h of aeration and incubation in Brine) an hour after the zebrafish spawn in the morning or an hour before putting them into the mating tank in the afternoon. After a month of domestication, zebrafish begin to spawn in pairs. Fish eggs were collected within 2 h after spawning and cultured at 28.5°C.

### 2.4 The toxicity of zebrafish

In this study, AB zebrafish embryos were randomly selected and divided into different groups with 6 embryos in each group on the 3rd day after fertilization. Each group was given different concentrations of KAE (1, 5, 10, 50, 100 μM), prednisolone (10, 15, 20, 25, 30 μM) or AAPH (10, 15, 20, 25, 30 mM) until 9 days after fertilization. The number of dead zebrafish in each group was counted to evaluate the toxicity of zebrafish embryos.

### 2.5 Experiment of zebrafish osteoporosis

To establish the model of osteoporosis, 3 dpf (day postfertilization) zebrafish in the DEX group, KAE group and ED group were exposed to 15 μM prednisolone to 5 dpf. At 6 dpf, mixed 15 μM prednisolone with KAE (1, 5, 10, 50, 100 μM) to KAE group, mixed 15 μM prednisolone with 50 μM ED to ED group, model group continued to give 15 μM prednisolone, blank control group was given the same amount of fish culture water, all groups changed 50% liquid every day to 9dpf. The juvenile fish were collected for bone mineralization analysis.

### 2.6 Fluorescence analysis of calcified vertebrae

Zebrafish larvae aged at 9 dpf were dyed with 0.2% calcein solution for 2 min. Then it was washed with water for 15 min (repeated three times). Zebrafish larvae were placed on a slide, and images of the vertebrae of zebrafish larvae were photographed using cellSens software and color digital fluorescence microscope (Japan Olympus co., ltd). The integrated density (IntDen) of calcein staining was measured by Image‐Pro Plus image analysis software Version 6.0 (Media Cybernetics). More than six zebrafish were used in each group. The relative fluorescence value is defined as the ratio of the measured value of each group to that of the blank group.

### 2.7 DCFH‐DA staining in zebrafish

Zebrafish embryos chosen through random selection and aged between 7 and 9 h (hpf), were grouped into 6‐hole culture plates. To evaluate the therapeutic effect of KAE on OS induced by AAPH, zebrafish embryos were induced by different concentrations of AAPH (10, 15, 20, 25, 30 mM). After 72 h of culture, zebrafish eggs hatched and reactive oxygen species production was evaluated by staining.

After conducting toxicity evaluations, several safe concentrations of KAE were selected for the purpose of evaluating its antioxidant properties. Embryos at 7–8 h post fertilization (hpf) were exposed to three distinct treatments: a control group with no treatment, a group induced with AAPH, and a group induced with AAPH and supplemented with KAE. Subsequently, 1 h after the preincubation period, AAPH (15 mM) induction solution was introduced to induce the oxidation of zebrafish. The production of reactive oxygen species (ROS) in juvenile zebrafish was measured 3 days after fertilization. Zebrafish larvae were stained with fluorescence in embryo culture medium containing DCFH‐DA (15 μg/mL). The fluorescence intensity of juvenile zebrafish was observed under fluorescence microscope after incubation for 1 h.

### 2.8 Network pharmacology

#### 2.8.1 Identification of KAE target

Input “kaempferol” into PubChem database (https://pubchem.ncbi.nlm.nih.gov/), retrieve and download the SDF format file and “standard smile count” of KAE. In addition, the Swiss Target Forecast (http://www.swisstargetprediction.ch/) and Pharmmapper database (http://lilabecust.cn/pharmmapper/index.html) is used to predict the potential protein associated with KAE through the “standard smile number.”

#### 2.8.2 Acquisition of disease targets

The targets of “OP” and “OS” were predicted by OMIM (https://www.omim.org/) Magnum TTD (https://db.idrblab.net/ttd/) and GeneCard database (https://www.genecards.org/) database.

#### 2.8.3 Determination of potential targets of drugs on diseases and construction of protein–protein (PPI) interaction network

Venny (https://bioinfogp.cnb.csic.es/tools/venny/) is used to obtain the intersection target between drug and disease, which is identified as the potential target of drug action on disease. The potential target is searched in the string database, the species is set to “Homo sapiens,” Score ≥0.4 is set as the minimum connection score, and finally the PPI network is obtained.

#### 2.8.4 GO and KEGG enrichment analysis

The DAVID platform was employed for performing gene ontology functional enrichment analysis and Kyoto Encyclopedia of Gene and Genome (KEGG) pathway enrichment analysis on the core targets. The resulting outcomes were stored and subsequently visualized through the utilization of the WeChat platform (https://www.bioinformatics.com.cn) to generate a bubble chart and bar chart.

### 2.9 Molecular docking

The pdb database (https://www.rcsb.org/), was utilized to retrieve and download the PDB format files of the proteins. The 3D conformation of KAE was obtained by downloading the SDF file from PubChem, which was subsequently converted into pdb format after energyoptimization using Chemdraw3D software. The pdb file obtained was subsequently transformed into pdbqt format using AutodockTools 1.5.6 software, functioning as the ligand for docking. The crystal structure of the target protein was obtained from the RCSB database, and the target protein was dehydrated, hydrogenated and docked with AutodockTools 1.5.6 software as the docking receptor. Ultimately, the docking process between the ligand and receptor was executed utilizing Autodock Vina software.

### 2.10 Molecular dynamics simulation

The starting configuration for the simulation of molecular dynamics was acquired through the findings derived from molecular docking. The Charmm36 force field and the TIP3P water model were used in the employment of Gromacs, a software designed for conducting dynamic simulations. To achieve equilibrium, sodium ions were introduced into a container of water. In the simulation process, the whole system minimizes energy to obtain an enhanced molecular configuration. Then, canonical ensemble method (NVT) and isothermal isobaric ensemble method (NPT) were used to balance the system at standard temperature and pressure, and molecular dynamics simulations were carried out for 100 ns. In the end, the evaluation simulation trajectory was evaluated using root mean square deviation (RMSD), root mean square fluctuation (RMSF), and rotation radius of the protein skeleton.

### 2.11 Cells culture

The RAW 264.7 mouse monocyte/macrophage cells were maintained in DMEM medium supplemented with 10% FBS and 1% (v/v) penicillin‐streptomycin solution and incubated at 37°C in 5% CO_2_ humidified air. RAW 264.7 cells were cultured with LPS (0.1 µg/mL) for a period of 72 h.

### 2.12 Tartrate‐Resistant acid phosphatase (TRAP) staining

To induce osteoclast differentiation, Log‐phase RAW 264.7 cells were divided into three groups: the model group, which was treated with lipopolysaccharide (0.1 μg/mL); The treatment group, which was exposed to lipopolysaccharide (0.1 μg/mL) and KAE (50, 40, 30 μM); And the control group, which received an equivalent volume of normal saline. After 72 h of culture, the cells underwent two rinses with phosphate buffered saline (PBS) and were treated with 4% paraformaldehyde (pH 7.4) at room temperature for 15 min. Tartrate‐resistant acid phosphatase (TRAP) staining was performed according to the instructions provided by the manufacturer. Osteoclasts were identified as TRAP positive multinucleated giant cells with two or more nuclei and were more dilated than RAW 264.7 cells and were counted under a microscope.

### 2.13 CCK8 analysis

RAW 264.7 cells in logarithmic phase were inoculated into 96‐well plate with a density of 1 × 10^4^ cells per well, and 200 μL cell culture medium was added to each well. The plate was subsequently placed in a CO_2_ incubator with a temperature of 37°C, 5% CO_2_, and constant humidity for a duration of 24 h to facilitate cell adhesion. Following this, KAE was introduced at concentrations of 50, 40, 30, 20, and 10 μM, and the cells were further incubated for an additional 24 h. Subsequently, 10 μL of cck8 reagent was added to each well and incubated for 2 h. The absorbance value at 450 nm was measured using a microplate reader. And then calculate the cell survival rate (%).

### 2.14 Effects of KAE on the contents of SOD, IL‐6, TRAP, NO, MAD, TNF‐α and GSH in RAW 264.7 cells

Following the aforementioned protocol, RAW 264.7 cells in the logarithmic growth phase were allocated into three distinct groups: the control group, model group, and administration group. Subsequently, a 72‐h stimulation was administered to induce osteoclasts, and the resulting supernatant was analyzed using an enzyme‐linked immunosorbent assay kit, in accordance with the provided instructions. This analysis aimed to determine the concentrations of SOD, MAD, GSH, TRAP, NO, TNF‐α, and IL‐6.

### 2.15 RT‐qPCR analysis

RAW 264.7 cells in logarithmic phase were divided into blank group, model group and administration group. Lipopolysaccharide (0.1 μg/mL) was added to the model group, 50 μg/mL and 0.1 μg/mL lipopolysaccharide were added to the treatment group, and the same amount of normal saline was added to the blank group. Cells were collected after 72 h of induction. Add 200 μL RNA isolater and store it in the refrigerator at −80°C. The cells were fully broken, added with 800 μL RNA isolater, centrifuged at a low speed for a short time, and left at room temperature for 2 min. Add 200 μL trichloromethane, fully mix 5 min, centrifuge 2 min, 12,000*g* at room temperature, transfer the transparent liquid in the upper layer of EP tube to another clean 1.5 mL EP tube at 4°C, 15 min; Add 500 μL isopropanol, fully mix, centrifuge 5 min, 12,000*g* centrifugation at room temperature, discard the supernatant at 4°C, 10 min; Add 75% ethanol 1 mL, centrifuge at 12,000*g* at 4°C for 5 min, rinse the precipitation twice. After discarding the supernatant, the EP tube retained with precipitation was inverted on clean filter paper and dried naturally; After proper drying, 50 μL of RNase‐free water was added to fully dissolve RNA; And the total RNA concentration was determined by ultraviolet microspectrophotometer; RNA was stored in a refrigerator at −80°C or used for cDNA synthesis. The total RNA was extracted by RNA extraction kit, and the first chain of cDNA was synthesized by Reverse Transcription kit according to the instructions of the kit. RT‐PCR was designed and synthesized by Shanghai Shenggong organism using primers. The sequence of primers is shown in Table 1. The RT‐PCR condition is 95°C, 5 min pre‐denaturation, 95°C, 20 s denaturation, 58°C, 45 s annealing, 72°C, 45 s extension, 72°C, 5 min re‐extension, a total of 40 cycles. RT‐PCR biology was repeated 3 times, and the mean value was taken as the result of biological repetition after removing the outliers.

**Table 1:** RT‐qPCR analysis of gene and associated base sequences.

Information	Gene	Sequence (5'to 3')	Length (bp)
NM_009652.3	M‐Akt1 (1)‐S	CTTCCTCCTCAAGAACGATGGC	118
	M‐Akt1 (1)‐A	TGTCTTCATCAGCTGGCATTGT	
NM_001410442.1	M‐Nfkb1‐S	GAGTCACGAAATCCAACGCAG	90
	M‐Nfkb1‐A	CGTCATCACTCTTGGCACAATC	
NM_010927.4	M‐NOS2 (1)‐S	TCACCTACCGCACCCGAGAT	169
	M‐NOS2 (1)‐A	GTACCAGGCCCAATGAGGATG	
NM_008610.3	M‐MMP2 (3)‐S	ACCTACACCAAGAACTTCCGATT	157
	M‐MMP2 (3)‐A	CAAAGACAATGTCCTGTTTGCAGA	
NM_008713.4	M‐eNOS (1)‐S	CTGCCACCTGATCCTAACTTGC	110
	M‐eNOS (1)‐A	AGCCCTTTGATCTCAATGTCGT	
NM_008366.3	M‐IL2 (2)‐S	AAACTAAAGGGCTCTGACAACACA	105
	M‐IL2 (2)‐A	GATGATGCTTTGACAGAAGGCTAT	
NM_001302531.1	M‐esr1‐S	TGTTGGATGCTGAACCGCC	224
	M‐esr1‐A	CCAGACGAGACCAATCATCAGA	
NM_001025395.2	M‐Src (1)‐S	AGATCACTAGACGGGAATCAGAGC	94
	M‐Src (1)‐A	GCACCTTTTGTGGTCTCACTCTC	

### 2.16 Statistical analysis

Experiments ought to be replicated a minimum of three times, and the outcomes should be presented as the mean value plus or minus the standard deviation (the mean ± SD). Single factor analysis of variance (ANOVA) was performed with SPSS 20.0 software, and statistical analysis was made for different measurement groups. The significant level of *p* < .05 is considered to indicate a statistically significant difference.

## 3 RESULT

### 3.1 Assessment of toxicity of KAE, prednisolone and AAPH to zebrafish

**Figure 1:**
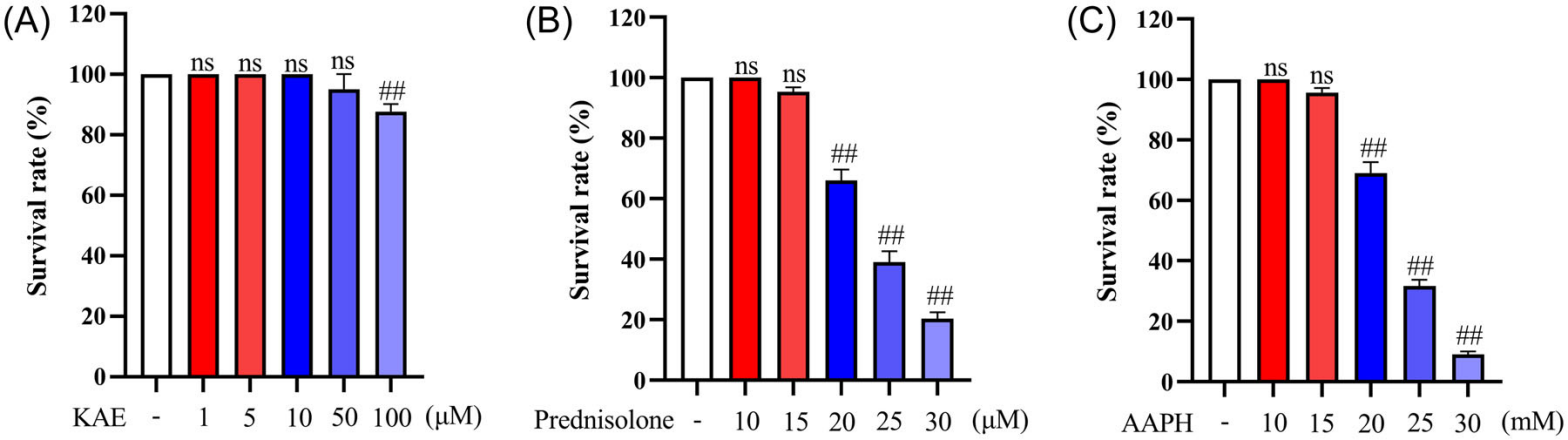
Effect of KAE, Prednisolone and AAPH concentration on the survival rates of zebrafish embryos. (A), (B) and (C) show the histogram of toxicity assessment of KAE, prednisolone and AAPH to zebrafish in turn. The abscissa represents the different concentrations of the drug and the ordinate represents the survival rate of zebrafish. Data were expressed as cell viability of the control group (the mean ± SD.), (*n* = 6) (^##^ *p* < .01 shows compared with the control group).

AAPH is an oxygen free radical producing reagent that is often used to induce OS in zebrafish. Prednisolone belongs to a glucocorticoid drug and long‐term use can induce OP. In this study, AAPH and prednisolone were used to establish models of OS and OP in zebrafish, respectively. Before the main study, an experiment was conducted to determine the toxicity of varying concentrations of AAPH and prednisolone on zebrafish embryos. This preliminary work is crucial for establishing safe and effective doses for the subsequent experiments. The results of the toxicity test showed that 48 h after administration, the concentration of KAE above 50 μM caused the death of zebrafish. Compared with the blank control group, the death number of zebrafish increased significantly at the concentration of 100 μM (*p* < .01). Therefore, zebrafish were further tested with 50 μM KAE. 20 μM prednisolone caused a large number of zebrafish deaths (*p* < .01). The toxicity of AAPH to zebrafish was concentration‐dependent and resulted in a large number of zebrafish death when the concentration was 20 mM (*p* < .01). Therefore, further experiments on zebrafish were carried out with 15 (μM or mM) prednisolone and AAPH Figure 1.

### 3.2 KAE ameliorates prednisolone‐induced zebrafish OP

**Figure 2:**
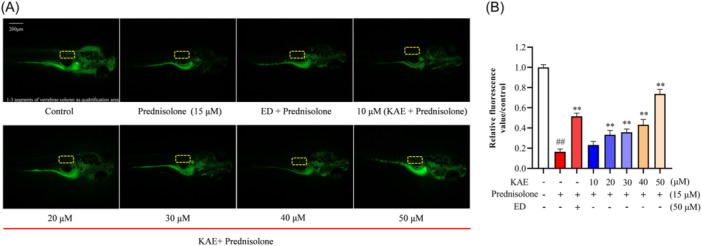
KAE interferes with Prednisolone‐induced OP calcein staining of zebrafish. (A) calcein staining of zebrafish. (B) relative fluorescence value of calcein staining under OP in zebrafish. Data were expressed as the mean ± SD. (*n* = 6). (^##^ *p* < .01 shows compared with the control group, and **p < .01 shows compared with the model group.).

Calcein staining showed that the bone fluorescence area of zebrafish in the model group was significantly lower than that in the control group, and the hypoplasia of the first three vertebrae could be clearly observed, indicating that the model of zebrafish OP was well established (Figure 2A). Compared with the model group, KAE treatment improved the OP of zebrafish. The fluorescence area of zebrafish bone increased significantly after treatment with different concentrations of KAE in a concentration‐dependent manner. At the concentration of 50 μM, KAE significantly improved prednisolone‐induced zebrafish OP (Figure 2B, *p* < .01).

### 3.3 KAE reduces AAPH‐induced OS in zebrafish

**Figure 3:**
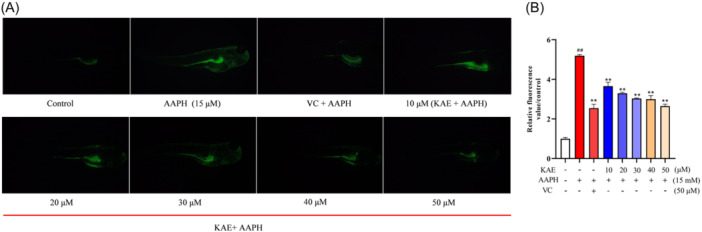
KAE interferes with AAPH‐induced OS DCFH‐DA staining of zebrafish. (A) OS DCFH‐DA staining of zebrafish. (B) relative fluorescence value of DCFH‐DA staining under OS in zebrafish. The abscissa values represent different concentrations of the drug. Data were expressed as the mean ± SD. (*n* = 6). (^##^ *p* < .01 shows compared with the control group, and **p < .01 shows compared with the model group.).

DCFH‐DA staining showed that the fluorescence area and reactive oxygen in zebrafish induced by AAPH were significantly increased, compared with the control group (Figure 3A, *p* < .01). The fluorescence values of VC and KAE in the treatment group were lower than those in the model group (Figure 3B, *p *< .01). KAE in all treatment groups decreased the level of reactive oxygen species in zebrafish in a concentration‐dependent manner. At the concentration of 50 μM, KAE had the best effect on reducing the level of reactive oxygen species in zebrafish.

### 3.4 Prediction of mechanism of KAE based on network pharmacology

**Figure 4:**
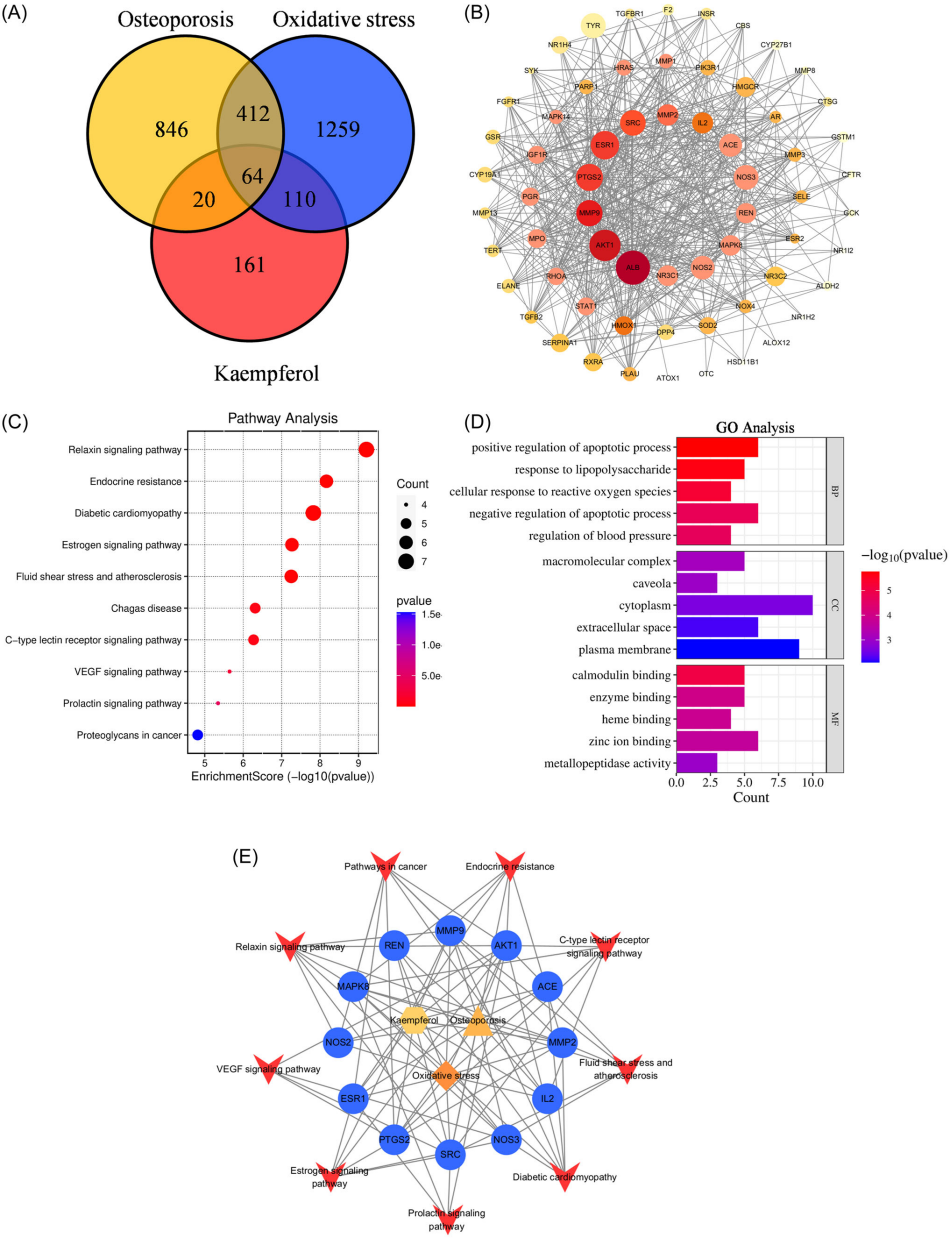
Network Pharmacology Analysis (A) Venn intersection target diagram. (B) PPI network interaction analysis. The targets are arranged in the order of CC values, the target size represents the BC value, and the color depth represents the DC value. (C) KEGG pathway enrichment analysis. (D) GO enrichment analysis. (E) “KAE‐Core Targets‐Pathways‐Disease” network analysis diagram.

1343 and 1845 targets related to OP and OS were obtained from CTD database, gene card and OMIM database. At the same time, 355 potential targets related to KAE were obtained from the Swiss target prediction database and PharmMapper database. 64 interacting proteins were identified (Figure 4A). The 64 intersection targets were sorted according to the center centrality (CC), betweenness centrality (BC) and degree centrality (DC) values calculated by Cytoscape software. The targets larger than the average of three values were considered to be the core targets of KAE in the treatment of OP and OS. Finally, a total of 14 core targets were selected (Figure 4B).

David data platform predicted 46 KEGG signal pathways. The top 10 signaling pathways with the most significant P values were Relaxin signaling pathway, endocrine resistance pathway, diabetic cardiomyopathy pathway, estrogen signaling pathway, fluid shear stress and atherosclerotic pathway, cancer pathway, Chagas disease pathway, C‐type lectin receptor pathway, vascular endothelial growth factor signaling pathway and prolactin signaling pathway (Figure 4C). Figure 4E shows the relationship between goals, diseases, and chemical composition. The 13 potential core targets of KAE in the treatment of OP and OS correspond to the first 10 rich signal pathways. 90 biological processes (BP), 12 cell compositions, and 30 molecular functions (MF) were identified (*p* < .01). According to the ascending order of *p* value, the top 5 BP, CC, and MF were selected and are shown in Figure 4D. BP was closely related to positive regulation of apoptotic process, response to lipopolysaccharide and cellular response to reactive oxygen species; CC was related to macromolecular complex, caveola and cytoplasm; and MF was related to calmodulin binding, enzyme binding and heme binding.

### 3.5 Simulated combination of KAE with core target

**Figure 5:**
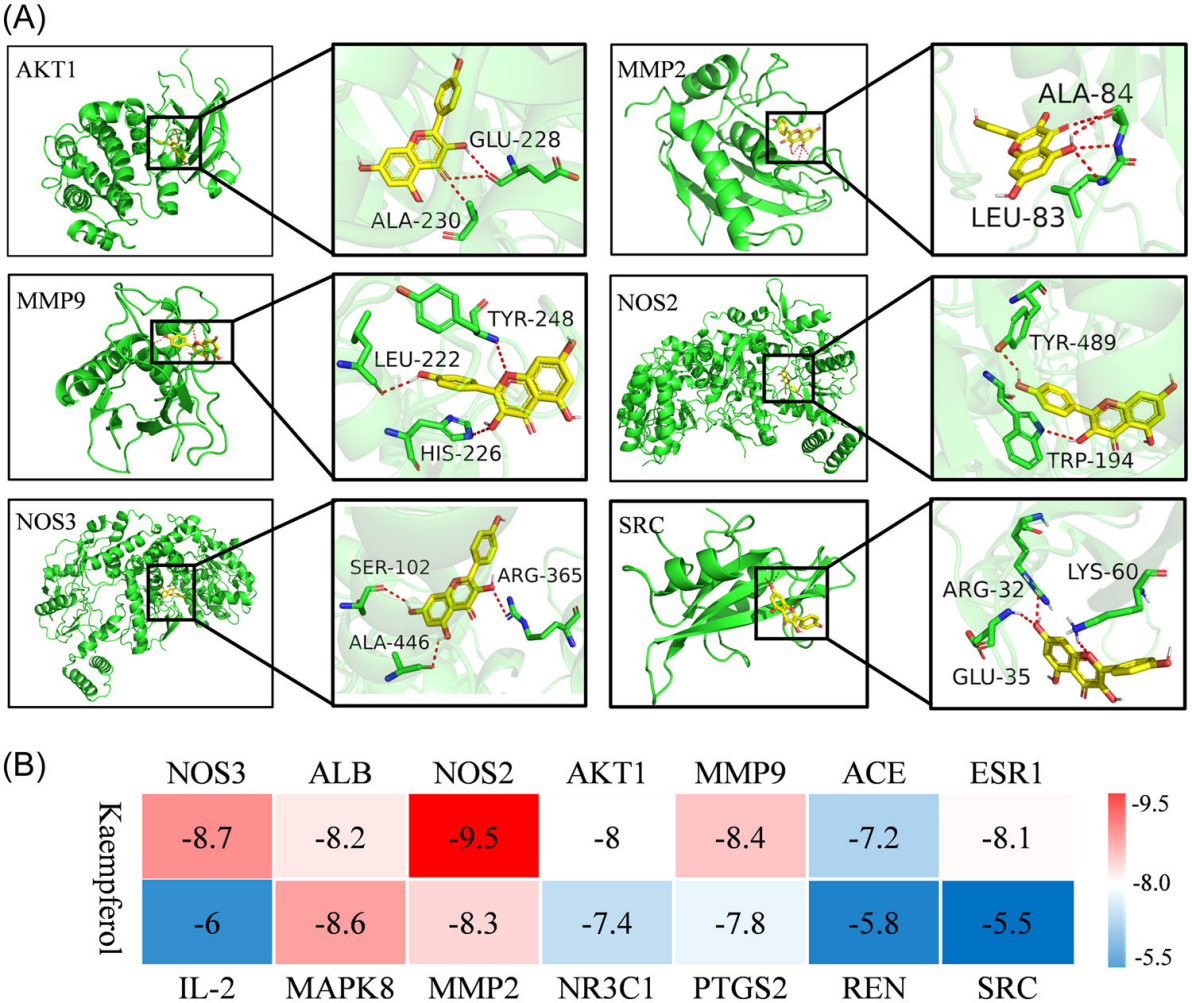
Visualization of docking between KAE and core target. (A) Interaction between KAE and amino acid residues of different core target proteins. (B) Docking binding energy of KAE with different core target molecules.

The results of molecular docking showed that the binding energies of KAE to the core target were >5.0 kj·mol^−1^ (Figure 5B), indicating that there was a strong interaction between KAE and the core target. In this study, proteins such as NOS2, NOS3, MMP2, MMP9, AKT1 and SRC are more critical, and the interaction between amino acid residues of these proteins and KAE is demonstrated by visualization (Figure 5A).

### 3.6 Molecular dynamics simulation of NOS2, MMP2, AKT1, SRC and KAE

Molecular dynamics simulation confirmed the durability of KAE binding to the target protein during the process of molecular docking. The analysis of the system employed the CHARMM force field and involved conducting 200 ns simulation samples to assess the structural stability of the composite target protein in molecular dynamics simulation. By analyzing the simulation trajectory, the root mean square deviation (RMSD), root mean square fluctuation (RMSF), and projection rotation radius were calculated.

**Figure 6:**
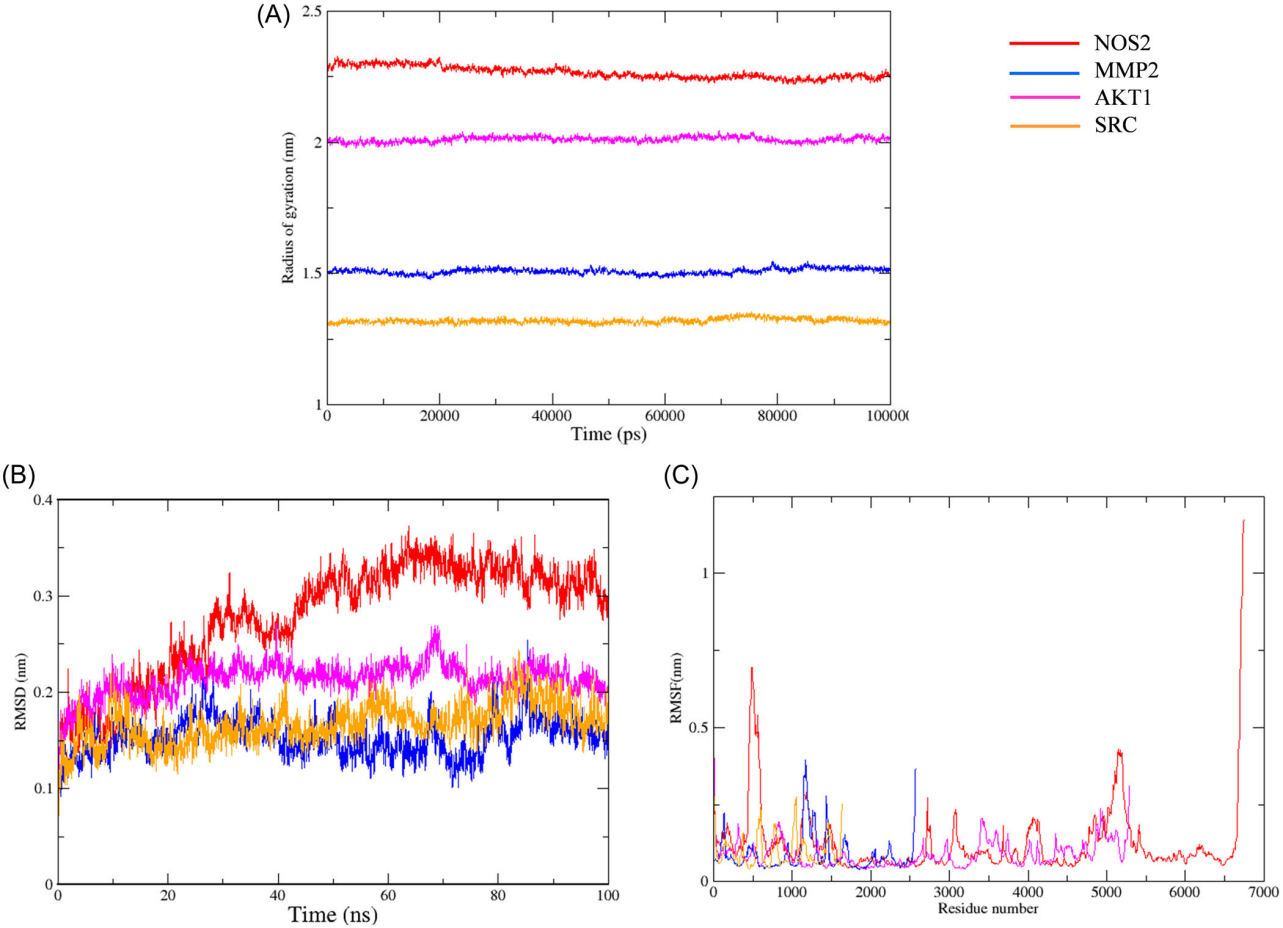
Molecular Dynamics Simulation. (A) RMSF Analysis. (B) Gyration Radius. (C) RMSD Analysis.

The stability of the system can be evaluated by employing the root mean square deviation (RMSD) as a standard measure. Within the simulation, the compactness of the protein structure is quantified by the radius of gyration. Analysis of Figure 6A demonstrates a diminished rotational radius of the protein, implying a denser protein structure and the establishment of a stable compound. Throughout the simulation, the RMSD value consistently maintains a low level, signifying a robust binding interaction between the ligand and the receptor, ultimately leading to the formation of a stable complex. Although the NOS2‐KAE complex exhibits more alterations compared to other systems, it remains within a stable range (Figure 6B). RMSD is utilized to describe the spatial variations of small chemical ligands in protein configurations. According to Figure 6C, the majority of the receptor components involved in the binding of KAE to the target protein persist within a consistent range. However, during the simulation of the 500, 500, 5000, and 6500 regions, the RMSF measurements of the NOS2‐KAE compound demonstrate significant fluctuations.

### 3.7 KAE inhibits osteoclast differentiation of RAW 264.7 cells induced by LPS

**Figure 7:**
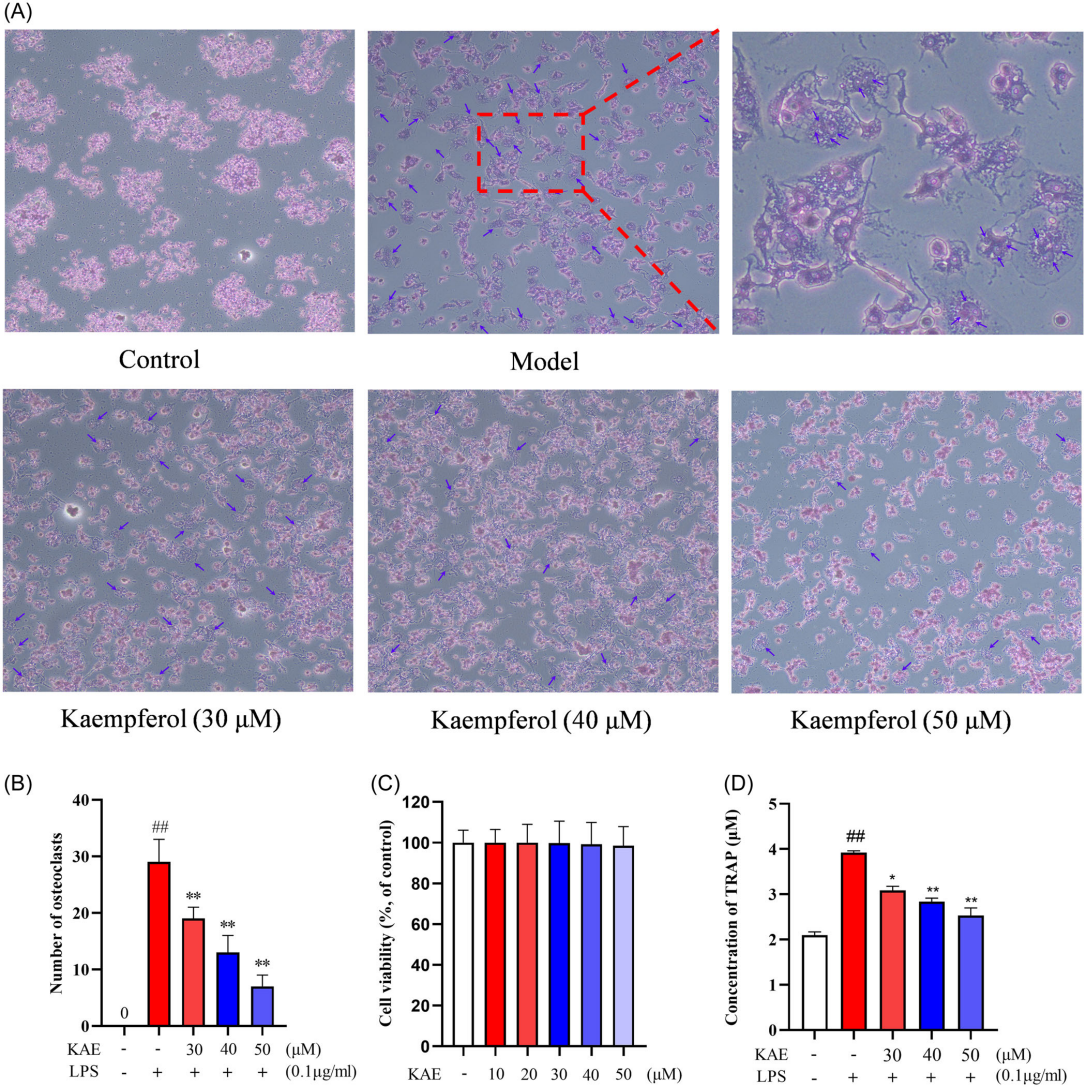
Effect of KAE on osteoclast differentiation of RAW 264.7 cells induced by LPS. (A) Tartrate resistant acid phosphatase (TRAP) staining of osteoclasts，multinucleated cells are considered to be osteoclasts (the number of nuclei ≥2). The blue arrow indicates the osteoclasts in each group. (B) The results of osteoclast count in each group (*n* = 3). (C) Effect of KAE on Cell Viability of Osteoclast Precursor. RAW 264.7 Cells (*n* = 6). RAW 264.7 cells were treated with or without KAE (10–50 µM) for 24 h. (D) TRAP level in osteoclasts differentiated from RAW 264.7 cells induced by LPS (*n* = 3). (^##^ *p* < .01 shows compared with the control group, *p < .05 shows compared with the model group, and **p < .01 shows compared with the model group.).

To investigate the impact of KAE on osteoclasts, RAW 264.7 cells were induced to differentiate into osteoclasts using LPS, both with and without the presence of KAE. Before this, CCK8 analysis was conducted to confirm that the inhibitory effect of KAE on osteoclast formation was not a result of its cytotoxicity towards the osteoclast precursor RAW 264.7 cells. The obtained data demonstrated that concentrations of 10–50 μM KAE exhibited no cytotoxic effects on the osteoclast precursors and did not impede their growth, as depicted in Figure 7C.

When KAE (30–50 μM) was used to interfere with osteoclast differentiation of RAW 264.7 cells induced by LPS, 72 h after adding LPS, osteoclast specific gene TRAP staining was performed on the induced cells. The data showed that LPS could directly stimulate osteoclast precursor RAW 264.7 cells to differentiate into osteoclasts. After induction of 0.1 μg/mL LPS for 72 h, a large number of dilated and multinucleated cells were observed in the model group. Compared with the model group, different concentrations of KAE caused RAW 264.7 cells to differentiate less into multinucleated giant cells (osteoclasts) (Figure 7A) and inhibited LPS‐induced osteoclast formation (Figure 7B) in a dose‐dependent manner. It is worth noting that 50 μM KAE significantly inhibited LPS‐induced osteoclast formation and decreased the level of osteoclast specific marker enzyme TRAP by 35.5% (Figure 7D) (*p* < .01).

### 3.8 KAE downregulates the expression of SOD, GSH, MDA, TNF, IL‐6 and NO induced by LPS in RAW 264.7 cells

**Figure 8:**
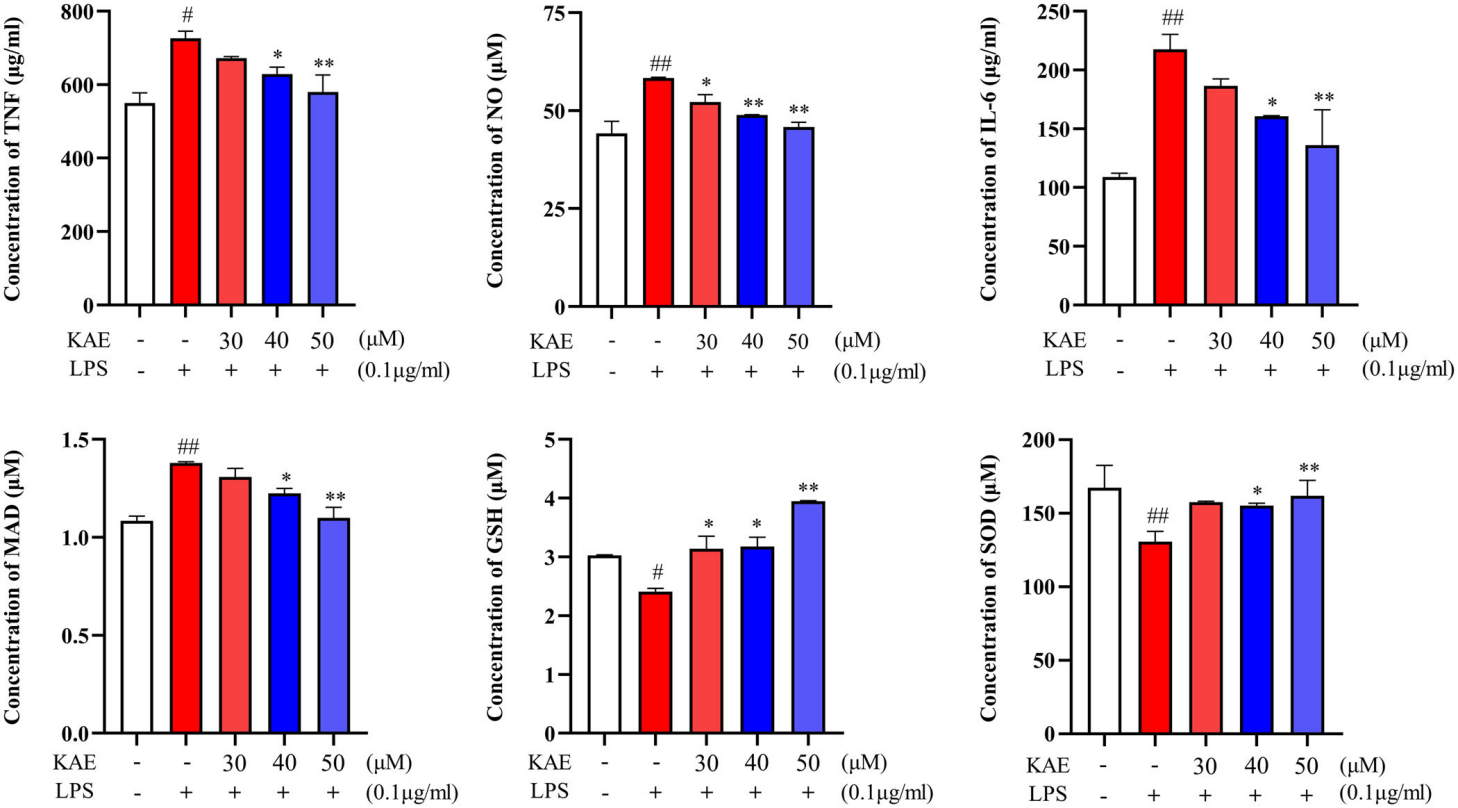
Detection of related cytokines in RAW 264.7 cells induced by LPS by KAE, (*n* = 3). (^#^ *p* < .05 shows compared with the control group, ^##^ *p* < .01 shows compared with the control group, *p < .05 shows compared with the model group and **p < .01 shows compared with the model group.).

The cytokine detection data demonstrated a significant increase in the level of MAD in RAW 264.7 cells after 72 h of LPS (0.1 μg/mL) administration, accompanied by a significant decrease in the levels of SOD and GSH (*p* < .01). These findings suggest the occurrence of OS in LPS‐stimulated RAW 264.7 cells. However, the administration of KAE demonstrated a concentration‐dependent reversal of these effects, as illustrated in Figure 8. Additionally, considering the regulatory influence of LPS‐induced inflammatory factors on RANKL, a pivotal factor in osteoclast differentiation, and the strong association between inflammatory factors and OS levels,^27^ the investigation also encompassed IL‐6, NO, and TNF. The findings indicated that KAE effectively decreased the levels of all three factors.^28^ Notably, a significant reduction in the levels of all examined factors, including TRAP, was observed with a concentration of 50 μM KAE. However, the effects of lower concentrations of KAE (30 μM) on TNF, IL6 and SOD were not different from those in the model group.

### 3.9 KAE downregulates osteoclast‐related genes in RAW 264.7 cells induced by LPS

**Figure 9:**
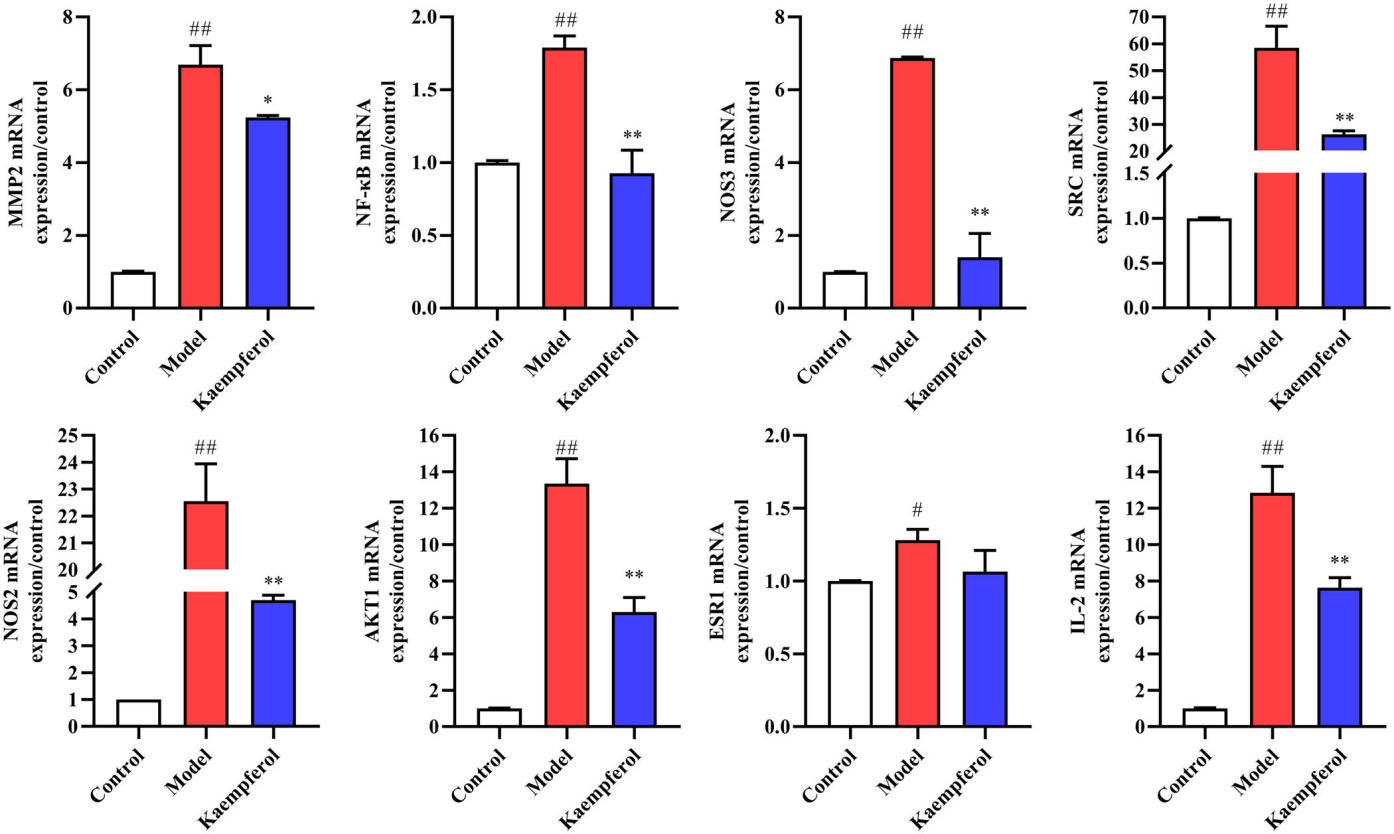
Effect of KAE on genes related to osteoclast formation in LPS‐induced RAW 264.7 cells, (*n* = 3). (^#^ *p* < .05 shows compared with the control group, ^##^ *p* < .01 shows compared with the control group, *p < .05 shows compared with the model group and **p < .01 shows compared with the model group.).

Quantitative RT‐PCR data showed that LPS at 0.1 μg/mL significantly upregulated the mRNA expression of MMP9, NF‐κB, NOS2, NOS3 and MMP‐9 genes during osteoclast differentiation. KAE significantly downregulated the mRNA expression of osteoclast‐related genes after 72 h treatment, and the inhibitory effect was dose‐dependent (Figure 9).

## 4 DISCUSSION

OP can be caused when bone homeostasis is disrupted. Many factors may contribute to this situation, including OS. KAE is a widespread natural antioxidant, so it is of great significance to develop KAE as a potential drug for the treatment of bone diseases. In this study, KAE has been shown to have a therapeutic effect on OP and OS in zebrafish. Network pharmacology predicts that the relaxin signal pathway obtained by enrichment of core targets is closely related to the role of KAE. Through the detection of related factors and quantitative PCR, it was proved that the SRC/NF‐κB‐AKT/NOS3 axis composed of core targets may be the key to the inhibition of osteoclasts by KAE.

Osteoclast‐mediated bone resorption is a key issue in the development of OP. In this study, Calcein staining showed that KAE had a therapeutic effect on OP, which may be achieved by promoting the proliferation and differentiation of osteoblasts or inhibiting the formation of osteoclasts. TRAP staining of osteoclasts showed that KAE could inhibit osteoclast differentiation of RAW 264.7 cells and significantly reduce the level of Trap in RAW 264.7 cells induced by LPS, which indicated that KAE could treat OP by inhibiting osteoclasts. At the same time, the levels of TNF, IL‐6, NO and MAD in RAW 264.7 cells decreased, while the levels of SOD and GSH increased in the KAE group, indicating that KAE slowed down OS. Inflammatory cytokines such as TNF and IL‐6 can regulate the production of RANKL, a key factor in osteoclast differentiation.^29^ GSH has been shown to inhibit the production of osteoclasts mediated by RANKL.^30^, ^31^ The overexpression of SOD inhibits the bone resorption of osteoclasts by reducing the level of ROS.^31^ When OS occurred, the levels of TNF, IL‐6, NO and MAD increased, while the levels of SOD and GSH decreased.^31^, ^32^ Therefore, in this study, KAE protects against OP by reducing OS and inhibiting osteoclast differentiation.

Network pharmacology predicts that the anti‐OP and OS effects of KAE are closely related to the relaxin signal pathway. Among the 6 core targets contained in this pathway, AKT1 regulates a variety of cellular processes, including metabolism, proliferation, cell survival and growth.^33^ PI3K/Akt pathway is part of the survival signal of cells facing OS.^34^ Meanwhile, AKT1 has been proved to be a key regulator of osteoclast differentiation and survival.^35^ NOS3 and NOS2 are key enzymes in the production of NO. The production of iNOS‐dependent NO helps to promote the survival of osteoclasts in TNF‐ α.^31^ At the same time, hypoxia and OS can also increase the production of NO, thus promoting the survival of osteoclasts.^36^ MMP2 and MMP9 belong to the matrix metalloproteinases (MMP) gene family, which can cut the components of extracellular matrix,^37^ expressing and participating in bone resorption.^38^ Studies have shown that OS can induce RUNX1 phosphorylation and SRC expression.^39^ SRC in osteoclasts is essential for osteoclast bone resorption.^40^ SRC deficient mice showed petrous osteopathy caused by inherent defects of osteoclasts.^41^ The results of molecular docking and molecular dynamics simulation also show that the binding of these core targets to KAE is good and stable, indicating that the enrichment results of the target pathway and the results of molecular docking confirm each other. Therefore, the targets closely related to OS and OP are screened from the core targets predicted by network pharmacology and are considered to be the key to the therapeutic role of KAE.

PCR results showed that KAE decreased the mRNA expression of AKT1, MMP2, NF‐κB, NOS2, NOS3, SRC, ESR1 and IL‐2 in RAW 264.7 cells induced by LPS. Rank is a key factor in stimulating osteoclast differentiation, and its expression is regulated by inflammatory cytokines such as TNF and IL‐2.^27^ The downregulation of TNF expression will affect the production of RankL.^42^ During osteoclast differentiation, Rank activates PI3K under the regulation of Src kinase activity,^27^ and PI3K then mediates the phosphorylation of NF‐κB by promoting Camp/PKA signal pathway.^43^ Just as early OS can activate NF‐κB,^44^ the activation of NF‐κB can promote the expression of NOS2, indirectly promoting the production of NO,^45^ indirectly promote the expression of MMP2/MMP9 genes, thus affecting bone balance.^46^, ^47^ Meanwhile, NF‐κB signaling pathway can also promote the differentiation and activation of osteoclasts and aggravate OP.^41^ Therefore, inhibition of NF‐κB can treat OP caused by OS.

At the same time, the expression of AKT1 can directly increase the phosphorylation of NOS3,^48^, ^49^ and then increase the production of NO and promote the expression of MMP2/MMP9 gene indirectly or directly by phosphorylating ERK.^50^, ^51^, ^52^, ^53^ Matrix metalloproteinases (MMPs) play an important role in osteoclast‐mediated bone resorption, while overexpression of MMP2/MMP9 can lead to matrix destruction and OP.

**Figure 10:**
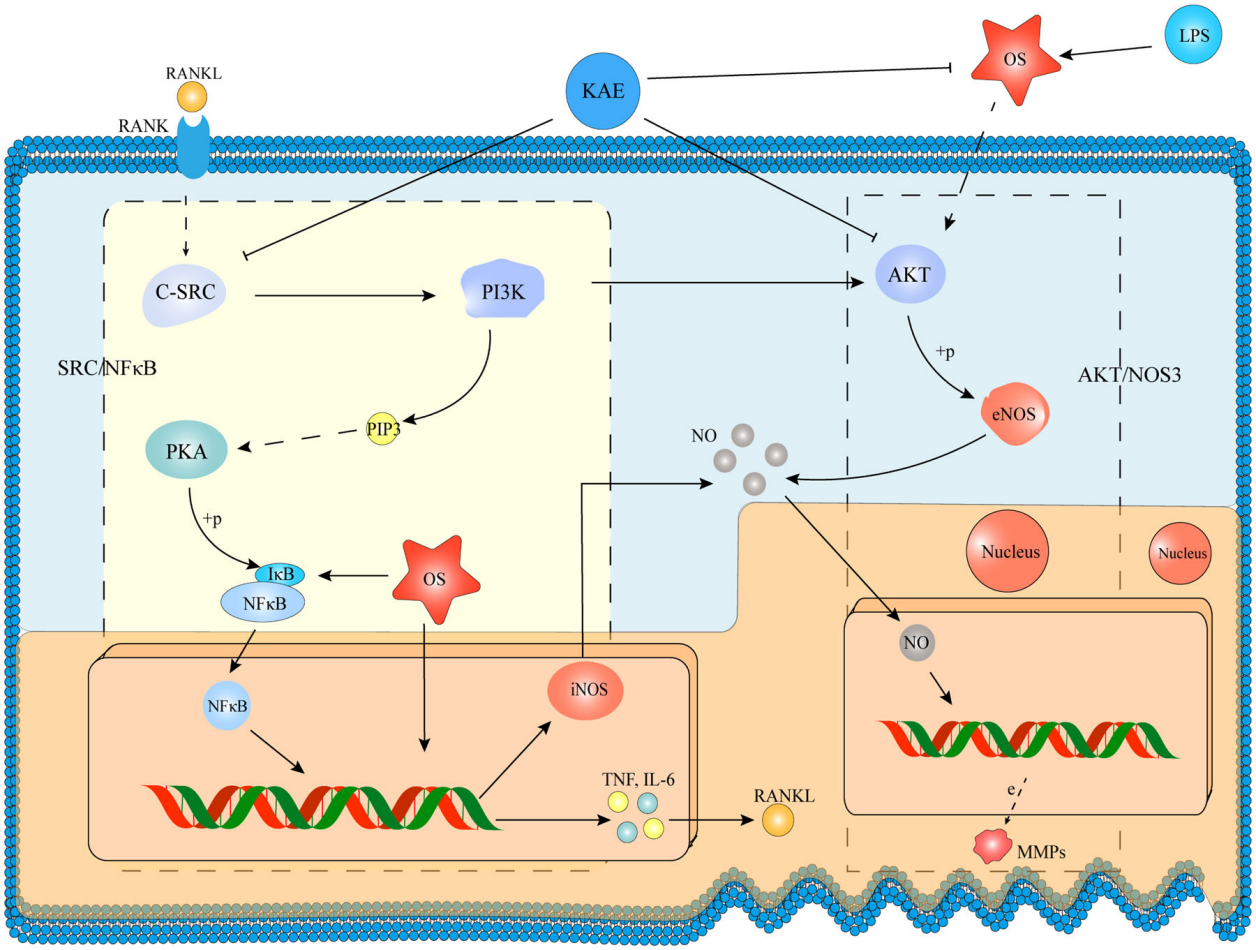
KAE attenuates LPS‐induced oxidative stress and inhibits osteoclast differentiation. OS stands for oxidative stress and KAE stands for KAE. This picture shows the mechanism of KAE in reducing oxidative stress and inhibiting the differentiation of RAW 264.7 cells into osteoclasts. The part inside the dotted line on the left shows the mechanism of SRC/NF‐κB. The part inside the dotted line on the right shows the mechanism of AKT/NOS3. In addition, some of the cell folds and multiple nuclei at the bottom of the picture indicate that osteoclasts are forming.

To sum up, downregulation of IL‐2, MMP, NOS3, NF‐κB, AKT1 and SRC by KAE can attenuate the effect of OS on these processes, thus reducing the effect of OS on osteoclast differentiation. Therefore, we conclude that KAE may treat OP by inhibiting SRC/NF‐κB‐AKT/NOS3 axis, reducing oxidative stress and inhibiting osteoclasts (Figure 10).

Notably, in other studies on the effects of KAE on osteoclasts,^54^, ^55^ KAE was found to inhibit osteoclast formation by suppressing autophagy induced by RANKL through downregulation of the expression of genes such as c‐Fos, NFATc1 and p62/SQSTM1. These studies demonstrated in detail the effects of KAE on autophagy‐related genes downstream of RANKL activation. Different from these studies, this study found that KAE attenuates OS and inhibits osteoclast generation by downregulating the genes downstream of RANKL, such as SRC, as well as by reducing the expression of OS‐induced genes, such as NFκB and NOS3. Despite the different research methods used, all of the above studies add to the mechanism of the osteoclastogenic inhibitory effect of KAE, confirming the effectiveness of KAE in inhibiting osteoclastogenesis and showing its potential application in the treatment of OP. In addition, other studies have shown that KAE has positive effects on osteoblasts. It promotes osteoblast survival,^56^ induces osteoblast differentiation through the WNT/β‐catenin signaling pathway,^12^ targeted stimulation of Krt‐14 protein contributes to matrix maturation and mineralisation of primary rat osteoblasts,^57^ and induces autophagy to promote osteoblast differentiation and mineralisation.^58^ These studies suggest that the regulation of bone homeostasis and bone formation by KAE results from the dual regulation of osteoclasts and osteoblasts, and therefore more biological studies are needed to investigate the regulation of bone homeostasis and bone formation by KAE in greater depth.

## 5 CONCLUSION

In summary, this work demonstrates that KAE inhibits osteoclast formation by inhibiting the SRC/NF‐κB‐AKT/NOS3 axis. It is suggested that KAE can treat OP by reducing oxidative stress and inhibiting osteoclasts. The results of this study explain the possible mechanism of KAE on osteoclasts and provide a new way for KAE to treat OP.

## AUTHOR CONTRIBUTIONS

**Jiaming Shen**: Investigation, validation, visualization, writing–original draft, writing–review & editing. **Chunjie Hu**: Investigation, validation, visualization, writing–review & editing. **Yuelong Wang**: Formal analysis, visualization. **Yiying Tan**: Formal analysis. **Xiaochen Gao**: Investigation. **Nanxi Zhang**: Investigation. **Jingwei Lv**: Formal analysis, data curation, visualization, supervision. **Jiaming Sun**: Formal analysis, data curation, supervision.

## CONFLICT OF INTEREST STATEMENT

The authors declare no conflict of interest.

## ETHICS STATEMENT

The animal study protocol was approved by the Laboratory Animal Ethics Committee of Changchun University of Chinese Medicine (protocol code: 2021519, date of approval: 19 November 2021) for studies involving animals.

## Data Availability

All relevant data are within the paper. If there is any other need for the journal, the authors will supply the relevant data in response to reasonable requests.
